# Sehverschlechterung und Blendungsempfindlichkeit nach Laservitreolyse

**DOI:** 10.1007/s00347-020-01221-3

**Published:** 2020-09-09

**Authors:** Larissa Lahme, Nicole Eter, Maged Alnawaiseh

**Affiliations:** Klinik für Augenheilkunde, Universitätsklinikum Münster, Domagkstr. 15, 48149 Münster, Deutschland

## Anamnese und Untersuchung

Es stellte sich eine 52-jährige Patientin zur Mitbeurteilung bei Blendungsempfindlichkeit v. a. abends, einer rechtsseitigen Hyperopie und einer Gangunsicherheit vor. Diese Symptome bestanden seit einer 5 Monate zuvor extern durchgeführten Laservitreolyse bei Glaskörpertrübungen. Vorbekannt war eine beidseitige leichte Myopie.

**Figure 1:**
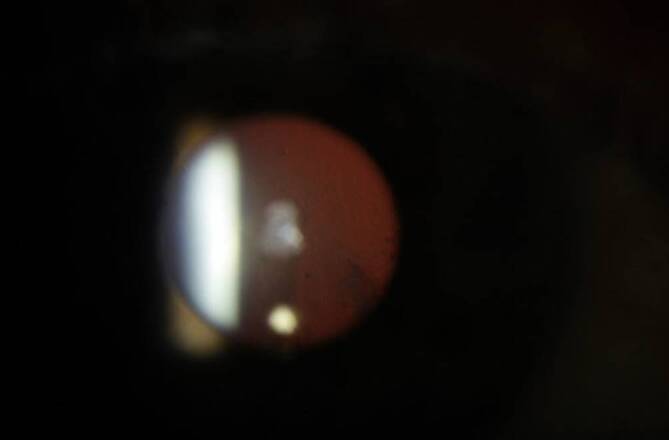

**Figure 2:**
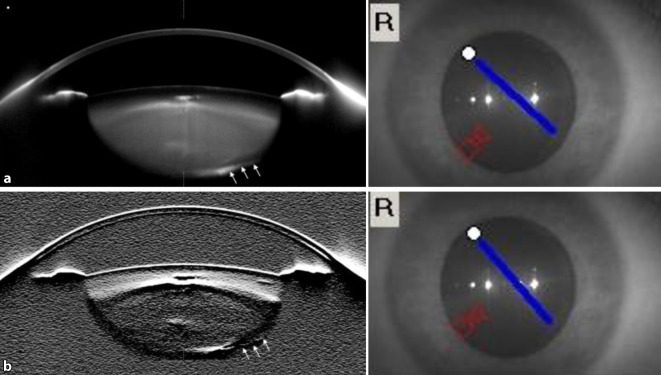

In der Untersuchung zeigte sich eine rechtsseitige Hyperopisierung mit einem rechtsseitigen Visus von 0,6 (Sphäre (S) +2,50 Zylinder (Z) −1,50 Achse (A) 14) und einem linksseitigen Visus von 0,8 (S −2,75 Z 0 A 0). Der intraokulare Druck war beidseits normoton. Rechtsseitig zeigte sich eine hintere Kapseltrübung mit Verdacht auf einen Kapselriss bei 4 und 8 Uhr bei einem sonst unauffälligen vorderen Augenabschnitt (Abb. 1). Linksseitig zeigte sich ein unauffälliger vorderer Augenabschnitt. Die Fundoskopie ergab beidseits einen unauffälligen Befund. Die Abb. 2 zeigt eine Scheimpflug-Tomographie-Aufnahme mit Darstellung des Hinterkapseldefekts.

## Wie lautet Ihre Diagnose?

**Diagnose:** Linsenverletzung mit Hinterkapseldefekt nach Laservitreolyse

## Therapie und Verlauf

Aufgrund der oben genannten Hinterkapseldefekte erfolgte eine Kataraktoperation mit anteriorer Vitrektomie und Implantation einer sulkusfixierten Linse. Es zeigte sich ein regelrechter intra- und postoperativer Verlauf. Die weiteren postoperativen Verlaufskontrollen erfolgten durch den niedergelassenen Augenarzt. Vier Wochen postoperativ stellte sich die Patientin erneut aufgrund eines seit einigen Tagen bemerkten lateralen Gesichtsfelddefektes am rechten Auge vor. Es zeigte sich eine rechtsseitige frische Amotio retinae ohne Makulabeteiligung. Es erfolgte eine unmittelbare operative Versorgung mittels Pars-plana-Vitrektomie und Gasendotamponade (C2F6 14 %). Am ersten postoperativen Tag zeigte sich eine leichte Fibrinreaktion in der Vorderkammer, sodass eine lokale Therapie mit Dexa-Gentamicin® Augensalbe (Wirkstoffe: Dexamethason 0,3 mg/g und Gentamicinsulfat 5,0 mg/g, Hersteller Ursapharm, Saarbrücken, Deutschland) und Inflanefran® forte Augentropfen (Wirkstoff: Prednisolonacetat, Hersteller Allergan, Frankfurt am Main, Deutschland) erfolgte. Hierunter zeigte sich eine rasche Befundbesserung. Bei einer Verlaufskontrolle 3 Wochen nach Entlassung zeigte sich ein zufriedenstellender postoperativer Befund mit einem bestkorrigiertem Visus von 1,0 beidseits.

## Diskussion

Der Glaskörper besteht aus extrazellulärer Matrix und zu 98 % aus Wasser [2]. Im Laufe des Lebens kommt es durch strukturelle Veränderungen aufgrund unterschiedlicher Prozesse wie Altersveränderungen, diabetischer Retinopathie, Entzündungen oder Myopie zu einer Verdichtung des Glaskörpers, was häufig zu der Wahrnehmung von Glaskörperfloatern führt [2, 3]. Glaskörperfloater beschreiben entopische Bilder, hervorgerufen durch Trübungen des Glaskörpers, die einen Schatten auf die Netzhaut werfen. Sie bewegen sich typischerweise bei Augen- und Kopfbewegung und verändern ihre Position innerhalb des Glaskörpers [2]. Insbesondere wenn sich diese im Bereich der optischen Achse befinden, können sie für den/die Patienten/-in sehr störend sein und die Lebensqualität reduzieren [2, 8]. Man unterscheidet zwischen primären Glaskörperfloatern, die v. a. durch degenerative Veränderungen hervorgerufen werden, und sekundären Glaskörperfloatern als Resultat einer okulären Inflammation [2]. Zwei mögliche Therapieformen bei Glaskörperfloatern stellen die Vitrektomie und die Yttrium-Aluminium-Granat(YAG)-Laser-Vitreolyse dar [3]. Bisher gibt es leider keine ausreichenden randomisierten Studien, die die Effektivität dieser beiden Methoden vergleichen [3]. Zusätzlich zu Linsenverletzungen kann es bei einer Laservitreolyse auch zu weiteren Komplikationen wie Verletzungen der Netzhaut und zu einem Offenwinkelglaukom kommen [1, 2]. Traumatische Hinterkapselverletzungen werden in der Regel mit einer Pars-plana-Vitrektomie, Lensektomie und Implantation einer Hinterkammerlinse behandelt. Zudem besteht die Möglichkeit einer primären epilentikulären IOL-Implantation kombiniert mit einer Pars-plana-Lentektomie [4, 6]. Interessant ist auch die Rolle des Femtosekundenlasers in der Behandlung solcher Fälle. Dieser kann insbesondere bei der Durchführung der Kapsulorhexis und der Linsensegmentation hilfreich sein [5] Eine Laservitreolyse sollte nur durchgeführt werden, wenn die Glaskörpertrübungen einen gewissen Abstand zur Netzhaut und zur Linse aufweisen und die Symptome chronisch vorhanden sind [2]. Ein routinemäßiger Einsatz wird aufgrund der oben genannten Komplikationen und der eingeschränkten Studienlage kontrovers diskutiert [7]. Weitere Studien, die die Sicherheit und die Langzeiteffektivität dieser Behandlungsmethode analysieren, sind daher notwendig.

## Fazit für die Praxis

Die Laservitreolyse stellt eine Möglichkeit zur Behandlung von symptomatischen Glaskörperfloatern dar. Bekannt sind neben der hier beschriebenen Linsenverletzung weitere Komplikationen wie Verletzungen der Netzhaut oder die Entstehung eines Offenwinkelglaukoms. Aufgrund dieser Risiken ist es sinnvoll, vor Durchführung einer Laservitreolyse eine optische Biometrie durchzuführen. In dem hier beschriebenen Fall zeigt sich trotz des komplizierten Verlaufs mit einer Verletzung der hinteren Linsenkapsel und einer Netzhautablösung nach erfolgter Kataraktoperation ein gutes Endergebnis.
